# A phase 2, open-label study of anti-inflammatory NE3107 in patients with dementias

**DOI:** 10.1097/MD.0000000000039027

**Published:** 2024-07-26

**Authors:** Jonathan Haroon, Kaya Jordan, Kennedy Mahdavi, Elisabeth Rindner, Sergio Becerra, Jean Rama Surya, Margaret Zielinski, Victoria Venkatraman, Dayan Goodenowe, Kaitlyn Hofmeister, Jeffrey Zhang, Clarence Ahlem, Christopher Reading, Joseph Palumbo, Bijan Pourat, Taylor Kuhn, Sheldon Jordan

**Affiliations:** a The Regenesis Project, Santa Monica, CA; b Synaptec Network, Santa Monica, CA; c Prodrome Sciences LLC, Temecula, CA; d Princeton Pharmatech, Princeton, NJ; e BioVie Inc., Carson City, NV; f Pourat MD, Beverly Hills, CA; g University of California Los Angeles, Los Angeles, CA.

**Keywords:** Alzheimer’s disease, amyloid beta, anti-inflammatory agent, cognitive function, dementia, glutathione, inflammation, magnetic resonance imaging, mild cognitive impairment, NE3107, phosphorylated tau

## Abstract

**Background:**

Alzheimer’s disease (AD) is a progressive, multifactorial, neurodegenerative disorder affecting >6 million Americans. Chronic, low-grade neuroinflammation, and insulin resistance may drive AD pathogenesis. We explored the neurophysiological and neuropsychological effects of NE3107, an oral, anti-inflammatory, insulin-sensitizing molecule, in AD.

**Methods:**

In this phase 2, open-label study, 23 patients with mild cognitive impairment or mild dementia received 20-mg oral NE3107 twice daily for 3 months. Primary endpoints assessed changes from baseline in neurophysiological health and oxidative stress (glutathione level) using advanced neuroimaging analyses. Secondary endpoints evaluated changes from baseline in neuropsychological health using cognitive assessments, including the 11-item Alzheimer’s Disease Assessment Scale-Cognitive Subscale (ADAS-Cog11), Mini-Mental State Examination (MMSE), Montreal Cognitive Assessment, Clinical Dementia Rating, Quick Dementia Rating Scale, Alzheimer’s Disease Composite Score, and Global Rating of Change (GRC). Exploratory endpoints assessed changes from baseline in neuroinflammation biomarkers (tumor necrosis factor alpha, TNF-α) and AD (amyloid beta and phosphorylated tau [P-tau]).

**Results:**

NE3107 was associated with clinician-rated improvements in cerebral blood flow and functional connectivity within the brain. In patients with MMSE ≥ 20 (mild cognitive impairment to mild AD; n = 17), NE3107 was associated with directional, but statistically nonsignificant, changes in brain glutathione levels, along with statistically significant improvements in ADAS-Cog11 (*P = *.017), Clinical Dementia Rating (*P = *.042), Quick Dementia Rating Scale (*P = *.002), Alzheimer’s Disease Composite Score (*P = *.0094), and clinician-rated GRC (*P < *.001), as well as in cerebrospinal fluid P-tau levels (*P = *.034) and P-tau:amyloid beta 42 ratio (*P = *.04). Biomarker analyses also demonstrated directional, but statistically non-significant, changes in plasma TNF-α, consistent with the expected mechanism of NE3107. Importantly, we observed a statistically significant correlation (*r* = 0.59) between improvements in TNF-α levels and ADAS-Cog11 scores (*P* = .026) in patients with baseline MMSE ≥ 20.

**Conclusion:**

Our results indicate that in this study NE3107 was associated with what appear to be positive neurophysiological and neuropsychological findings, as well as evidence of improvement in biomarkers associated with neuroinflammation and AD in patients diagnosed with dementia. Our findings are consistent with previous preclinical and clinical observations and highlight a central role of neuroinflammation in AD pathogenesis.

## 1. Introduction

Chronic neuroinflammation is thought to play a significant role in the progression of neurodegenerative disorders, such as Alzheimer’s disease (AD).^[1–3]^ AD pathologies, amyloid beta (Aβ), and phosphorylated tau (P-tau), are thought to trigger pro-inflammatory responses to drive chronic inflammation, which in turn may contribute to further accumulation of Aβ and P-tau, accelerating neurodegeneration.^[1,4,5]^ Tumor necrosis factor-alpha (TNF-α), a key inflammatory mediator, is capable of inducing oxidative stress through the production of reactive oxygen species and reduction of glutathione, an important antioxidant.^[6–8]^ Additionally, neuroinflammatory mediators can disrupt insulin signaling via serine phosphorylation of insulin receptor substrate-1, leading to insulin resistance, which can subsequently contribute to increased Aβ peptide 42 (Aβ42) production and neuritic plaques, impaired glucose metabolism, and, eventually, cognitive decline.^[5,9–13]^ Therefore, anti-inflammatory and insulin-sensitizing drugs are being explored for their potential to slow AD progression and the associated cognitive decline.^[4,5,14]^

NE3107 is a chemically modified analog of a physiologic human adrenal sterol, β-androstenetriol, and is an orally bioavailable, metabolically stable, blood-brain barrier–permeable molecule currently being investigated for its potential therapeutic effects in neurodegenerative disorders, including AD.^[5]^ NE3107 is thought to bind the inflammatory mediator, extracellular signal-regulated kinase (ERK), and selectively inhibit inflammation-stimulated ERK, nuclear factor kappa B (NF-κB), and TNF-α signaling, without compromising any homeostatic functions of ERK.^[5]^ In pre-clinical studies, NE3107 was shown to modulate several inflammatory mediators associated with cognitive decline, such as ERK, NF-κB, TNF-α, and interleukin 1 beta (IL-1β), as well as monocyte chemoattractant protein-1 and its receptor, chemokine C-C motif receptor 2 (CCR2).^[5,15,16]^ In humans, NE3107 was associated with decreased C-reactive protein, an inflammatory biomarker implicated in AD.^[5]^ Given the direct influence of pro-inflammatory mediators on insulin signaling, NE3107 has been proposed to possess insulin sensitizing activity.^[5]^ Results from phase 1 and 2 clinical trials indicated that NE3107 improved insulin sensitivity and restored metabolic homeostasis in patients with impaired glucose tolerance and type 2 diabetes and inflammation.^[17,18]^ Importantly, NE3107 was well tolerated and had a favorable safety profile across 6 clinical trials.^[5]^

In the present, exploratory investigation, NE3107-treated patients were evaluated for changes in advanced neuroimaging, cognitive performance tests, and central nervous system (CNS) biomarkers of AD, as well as improvements in inflammatory serological and metabolic parameters. Based on NE3107’s anti-inflammatory mechanism of action and immunomodulatory effects, it was hypothesized that 20 mg of NE3107 administered twice daily (BID) in patients with mild cognitive impairment (MCI) or dementia would lead to (1) changes in neuroimaging consistent with increased brain glutathione (indicating decreased oxidative stress) and enhanced arterial perfusion, as well as increased functional connectivity (FC) of the default mode network (DMN), nucleus basalis of Meynert (NBM), and hippocampal (HC) networks, (2) improvements in neurocognitive functioning, and (3) reduction in biomarkers associated with inflammation (TNF-α) and AD pathology (Aβ42 and P-tau), as well as improvements in biomarkers associated with glucose metabolism.

## 2. Methods

### 2.1. Patient recruitment

This study (Clinical Trial Identifier #NCT05227820) was reviewed and approved by Advarra Institutional Review Board (Protocol Identifier: Pro00058626). The most recent approval number is CR00403211. Twenty-three patients were enrolled from Los Angeles neurology clinics. All patients provided written, signed informed consent.

Participants were recruited through a network of neurology clinics in Los Angeles. When patients expressed interest, study staff informed participants and their caregivers about the protocol, potential risks and benefits, follow-up procedures, and expenses. Participants and caregivers were given the opportunity to ask any questions they might have had after an extensive perusal of the informed consent. To ensure commitment and attention to follow-up procedures, caregivers were involved in the consenting process. Each participant gave their written, signed consent.

Inclusion criteria required patients to (1) have a clinical diagnosis of cognitive decline due to degenerative dementia, (2) be aged from 50 to 89 years old, and (3) have a Quick Dementia Rating Scale (QDRS) score ranging from 1.5 to 12.5 with a converted Clinical Dementia Rating (CDR) score of 0.5 (MCI) to 1 (mild dementia). Diagnoses were established by the principal investigator who is a board-certified neurologist.

Patients were excluded from consideration if any of the following criteria were met: contraindication to lumbar puncture, contraindication to magnetic resonance imaging (MRI), women who were pregnant or could have become pregnant, diagnosis of a reversible cause of cognitive impairment that explained the clinical status entirely (e.g., depression, hypothyroidism), advanced stages of terminal illness or active cancer requiring chemotherapy, or a history of breast cancer. Women with child-bearing potential who were not willing to use a double-barrier birth control method were excluded from enrollment, as were males not willing to use a double-barrier birth control method with female sex partners with child-bearing potential. Individuals with hepatic or renal impairment were excluded from enrollment as well.

### 2.2. Study procedures

Patients received 20-mg oral NE3107 (BioVie Inc., Encinitas, CA) BID approximately 12 hours apart for 3 months. The dose was kept stable for the duration of the study intervention and was the same for all patients.

Following confirmation of the inclusion and exclusion criteria, patients underwent baseline testing to include the following: advanced MRI of the brain, lumbar puncture, apolipoprotein E genotyping, epigenetic analysis, cognitive testing, and serological analysis.

### 2.3. Study endpoints

Primary endpoints involved a comparison of neurophysiological health as evaluated by multimodal brain MRIs obtained at baseline and post intervention termination (3 months). Specifically, multimodal MRI endpoints included: (1) an increase or stabilization in glutathione levels (as measured by magnetic resonance spectroscopy, MRS) compared to baseline; patients with cognitive decline often show a characteristic change in MRS or volumetric evaluation compared to age-matched controls.^[19]^ (2) Enhancement of arterial perfusion compared to baseline (as quantified by arterial spin labeling, ASL); patients with cognitive decline often have decreased perfusion in temporal-parietal or frontal regions of the brain, as detected with ASL perfusion.^[19]^ (3) Increased FC of the NBM with both hippocampi as well as between both hippocampi compared to baseline, as visualized by seed analysis of blood-oxygen-level-dependent (BOLD) imaging. (4) Increased neurovascular coupling as visualized by BOLD imaging compared to baseline. (5) Stabilized and/or improved dendritic density compared to baseline (as measured by diffusion tensor imaging, DTI-NODDI).

Secondary endpoints assessed several neuropsychological testing measures and included the QDRS, CDR score as estimated by the QDRS, the 11-item Alzheimer’s Disease Assessment Scale–Cognitive Subscale (ADAS-Cog11), Mini-Mental State Examination (MMSE), Montreal Cognitive Assessment (MoCA), AD Composite Score (ADCOMS), and Global Rating of Change (GRC). Exploratory endpoints included a longitudinal comparison of metabolic and serological analyses, specifically, measures of inflammation, AD pathology, and glucose metabolism.

### 2.4. Primary endpoint: neuroimaging

Each prospective patient underwent advanced magnetic resonance neuroimaging during the study screening period, prior to treatment administration, to obtain baseline measurements. The MRI included volumetric analysis of the HC and lobes of the brain, perfusion values (relative cerebral blood flow, rCBF) quantified by ASL, neuronal FC (visualized by BOLD imaging), and MRS of the precuneus.

Patient scans were conducted at a single imaging center in Beverly Hills, CA. All magnetic resonance data were quality-controlled prior to being processed and reviewed. Post-processing was done using the Functional Magnetic Resonance Imaging of the Brain Software Library (https://fsl.fmrib.ox.ac.uk/fsl/fslwiki/) with standardized parameters for all patients. Identical follow-up MRI scans were done at the same imaging center where baseline scans were conducted. MRS post-processing was completed using Tarquin analysis (https://tarquin.sourceforge.net/). During the MRI data processing and pre-processing phase, there were several quality checks employed to ensure accuracy. These checks involved image and alignment co-registration, bias field correction, and skull stripping. Additionally, there were functional MRI (fMRI) quality checks specifically focused on motion correction to guarantee reliable results for the functional data, including removal of movement artifact in BOLD imaging and ASL.

### 2.5. Blinded review

fMRI data were qualitatively judged by 2 trained, blinded investigators: a board-certified neurologist with expertise in neuroradiology and a neuropsychologist with expertise in fMRI and subsequent statistical analysis. In the event of disagreement between raters, data were subjected to additional review and ultimately scored by consensus.

Baseline scans were rated as abnormal or normal according to the following criteria: for perfusion, measured by rCBF, a 30% decrease in signal intensity in the temporal, parietal, or occipital lobes (in at least 1 hemisphere) was scored as abnormal; for BOLD, seed-based FC, a cluster >1 cm^3^ shown on a statistical map would be scored as abnormal for the following connectivity patterns: for a seed placed in the NBM, abnormal regions for cluster included contralateral and/or ipsilateral NBM (beyond the area of the seed), inferior frontal lobe, temporal lobe, and basal forebrain; for a seed placed in the HC, abnormal regions for cluster included contralateral and/or ipsilateral HC (beyond the area of the seed) and anterior mid-temporal lobe; for a seed placed in the precuneus, abnormal regions for cluster included prefrontal midline region (excluding the interhemispheric fissure) and parietal lobe. Follow-up scans were rated as improved: no longer meets criteria for abnormality; declined: previously “normal” scan met criteria for abnormality at follow-up or abnormality became more extensive in an abnormal patient; or stable: no significant change between baseline and follow-up scans.

### 2.6. Group analysis

After preprocessing (motion correction, artifact removal, spatial smoothing) the resting-state fMRI, FC analysis was performed with a seed set for the following regions of interest (ROIs): the precuneus (Fig. S1A, Supplemental Digital Content, http://links.lww.com/MD/N250), the left and right NBM, and the left and right HC (Fig. S1B, Supplemental Digital Content, http://links.lww.com/MD/N250). The NBM seeds were drawn in Montreal Neurological Institute (MNI) standard space and transformed to functional space for first-level analysis, the HC seed was taken from the Functional Magnetic Resonance Imaging of the Brain Software Library subcortical segmentation tool (Functional Magnetic Resonance Imaging of the Brain’s Integrated Registration and Segmentation Tool), and the precuneus was taken from MNI standard space as well. The HC and NBM seeds were also in probabilistic tractography to map out the white matter connections. Glutathione concentrations in the precuneus were measured using MRS analysis (Fig. S1C, Supplemental Digital Content, http://links.lww.com/MD/N250). Group analysis was performed in standard MNI space using a mixed effects model with age and sex as additional covariates.

### 2.7. Secondary endpoints: neuropsychological testing

Secondary endpoints also evaluated changes in cognitive functioning using the QDRS, CDR score (estimated by the QDRS), ADAS-Cog11 (scored from 0–70), MMSE, MoCA, and ADCOMS. The QDRS form consists of 10 categorical questions, 5 of which are cognitive and 5 of which are functional. Each question has 5 detailed answers, each of which represents an impairment level of 0 (normal), 0.5, 1, 2, or 3 (severe impairment). The person designated as the caregiver at baseline consented to do the QDRS interview at completion to maintain consistency. Total QDRS scores were converted to CDR scores ranging from 0 (normal aging), 0.5 (MCI), 1 (mild dementia), 2 (moderate dementia), and 3 (severe dementia).^[20]^ The ADAS-Cog11 consists of 11 components that assess spoken language, comprehension, word-finding difficulties, object/figure naming, command following, constructional praxis, ideational praxis, orientation, word recognition, and test direction recall.^[21]^ The MMSE is a 30-point test that assesses cognitive ability.^[22]^ Specific tasks that measure orientation, attention, memory, language, and visuospatial abilities are part of the MMSE. The MoCA was used to assess general cognitive function.^[23]^ It measures immediate and delayed memory, attention, language, visuospatial construction, orientation to time and place, and frontal executive skills. Scores on the MoCA scale range from 0 to 30; 26 or more is regarded as reflecting normal cognitive status.^[24]^ To minimize practice effects between baseline and completion testing, different MoCA iterations were administered. ADCOMS was used to ascertain treatment effects and is composed of 4 ADAS-Cog items, 2 MMSE items, and all 6 items of the CDR—Sum of Boxes.^[25]^

Patients, clinicians, and caretakers also reported a GRC upon study completion.^[26]^ The GRC was assessed using an 11-point scale to track changes in a patient’s conditions, abilities, and overall sense of well-being, where 0 indicated “no change,” +5 indicated “significantly better,” and −5 indicated “significantly worse.”

### 2.8. Exploratory endpoints: biomarkers

Secondary endpoints evaluated changes in glucose metabolism markers (hemoglobin A1c and urinary glucose levels), changes in inflammatory markers (high-sensitivity C-reactive protein, erythrocyte sedimentation rate, and transforming growth factor beta), and changes in levels of several cytokines (TNF-α, IL-1β, IL-6, and IL-12).

All patients underwent apolipoprotein E swab testing and a lumbar puncture to assess Aβ42 and Tau proteins for AD spectrum. The lumbar puncture was performed at baseline and after completion of the study protocol. Table 1 summarizes all biomarkers assessed and the assays used. These assessments were carried out by commercial laboratories. Additional procedures included blood and serum samples collected at baseline and completion to ensure patient safety and the effect of NE3107 on the laboratory values listed above. Clinical laboratory tests for safety were collected at baseline, 2 weeks, 4 weeks, 8 weeks, and completion, while laboratory tests for efficacy were collected at baseline and completion. Epigenetic analyses are in progress and will be reported at a later time.

**Table 1:** Biomarkers assessed in the trial.

Biomarker	Assay
HbA1c	Enzymatic assay
Urinary glucose levels	Reagent strip and light microscopy
hsCRP	Immunoturbidimetric assay
ESR	Westergren
TGF-β	Enzyme Linked Immunosorbent Assay (ELISA)
IL-1 β	Enzyme Linked Immunosorbent Assay (ELISA)
IL-6	Immunoassay
IL-12	Immunoassay
TNF-α	Immunoassay
APOE	Single nucleotide polymorphism (SNP) genotyping
AB42/tau ratio	Electrochemiluminescent Immunoassay (ECLIA)

APOE = apolipoprotein E, HbA1c = hemoglobin A1c, TGF-β = transforming growth factor beta.

### 2.9. Statistical analysis

Paired sample t-tests were used for statistical analyses of the secondary endpoints, including biomarkers and neuropsychological testing, which compared the change from baseline at the post-intervention termination follow-up (3 months after baseline) using a level of significance of 0.05. Software for statistical analyses included SAS (version 9.4; SAS Institute Inc., Cary, NC) and R (version 4.0). Since this was an exploratory investigation, the study was not formally powered for any endpoint. The protocol was designed to examine MCI and mild dementia. Since the conventional CDR sum of boxes was not used to determine the Global CDR score, the standard MMSE was used to define mild (MMSE ≥ 20) versus moderate (MMSE < 20) impairment. For exploratory analyses, *P* values were not adjusted for multiple determinations.

## 3. Results

**Figure 1.**
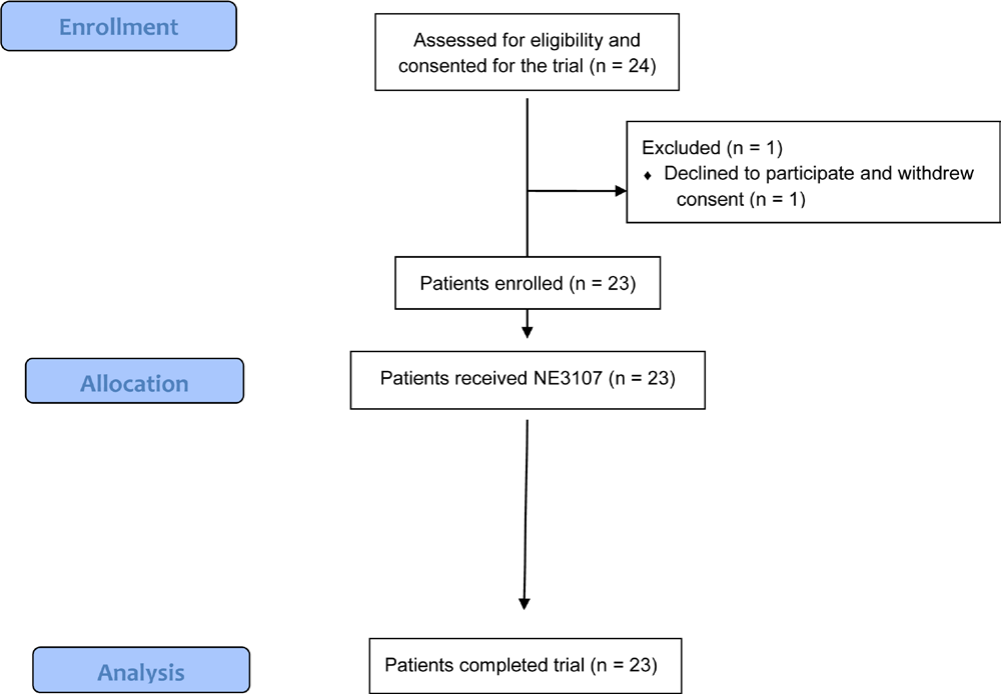
Flowchart of the study selection process.

Patients were enrolled from January 2022 through April 2022. A total of 23 patients completed the study and received NE3107 over the course of 3 months (Fig. 1). At baseline, all patients had a QDRS score ranging from 1.5 to 12.5, with a converted CDR score of 0.5 (MCI) to 1 (mild dementia). A description of patient demographics, including patient baseline demographics and dispositions, can be found in Table 2.

**Table 2:** Baseline characteristics.

Characteristic	All patients (N = 23)
Age, mean (SD), years	71.1 (9.50)
*Gender, n* (%)	
Female	16 (70)
Male	7 (30)
*Family history, n* (%)	
AD	5 (22)
AD, dementia, unspecified etiology	2 (9)
AD, PD	1 (4)
Dementia, unspecified etiology	4 (17)
PD	1 (4)
QDRS score, mean	5.07
*CDR score, n* (%)	
0.5	18 (78)
1	5 (22)
*MMSE, n* (%)	
≥20 (MCI to mild AD)	18 (78)
<20 (moderate AD)	5 (22)
*APOE status, n* (%)	
ε2/ε3	2 (9)
ε2/ε4	1 (4)
ε3/ε3	9 (39)
ε3/ε4	10 (44)
ε4/ε4	1 (4)

AD = Alzheimer’s disease; *APOE* = apolipoprotein E; CDR = Clinical Dementia Rating; MCI = mild cognitive impairment; MMSE = Mini-Mental State Examination; PD = Parkinson disease; QDRS = Quick Dementia Rating System.

### 3.1. Primary endpoint: neuroimaging

#### 3.1.1. Blinded review

Functional MRI modalities, including rCBF and BOLD, seed analysis of the NBM, HC networks, and DMN, were subjected to blinded review by 2 independent clinicians with a predetermined rubric. The average inter-rater reliability for all functional MRI modalities was 96%. The DMN had the highest abnormality at baseline with 86% (19/22) of patients demonstrating abnormality in DMN FC. This was followed by the HC networks where 77% (17/22) of patients demonstrated an abnormality in the left HC network, 82% (18/22) in the right. The greatest improvement was seen in the HC networks, with 45% (10/22) of patients demonstrating improved FC at follow-up for the left HC, 54% (12/22) for the right (Table 3). DTI-NODDI analyses are still in progress.

**Table 3:** Clinician-rated scores of functional magnetic resonance imaging at baseline and follow-up.

Functional MRI modality	Follow-up		
n (%)	Declined n (%)	No change n (%)	Improved n (%)
*rCBF*			
Total, 22 (100%)	2 (9%)	17 (77%)	3 (14%)
Normal, 6 (27%)	1 (17%)	5 (83%)	0 (0%)
Abnormal, 16 (73%)	1 (6%)	12 (75%)	3 (19%)
*BOLD left NBM*			
Total, 22 (100%)	2 (9%)	12 (55%)	8 (36%)
Normal, 14 (64%)	2 (14%)	10 (72%)	2 (14%)
Abnormal, 8 (36%)	0 (0%)	2 (25%)	6 (74%)
*BOLD right NBM*			
Total, 22 (100%)	3 (14%)	12 (55%)	7 (32%)
Normal, 13 (59%)	3 (23%)	9 (69%)	1 (8%)
Abnormal, 9 (41%)	0 (0%)	3 (33%)	6 (67%)
*BOLD DMN*			
Total, 22 (100%)	6 (27%)	9 (41%)	7 (32%)
Normal, 3 (14%)	1 (33%)	2 (67%)	0 (0%)
Abnormal, 19 (86%)	5 (26%)	7 (37%)	7 (37%)
*BOLD left HC network*			
Total, 22 (100%)	5 (23%)	7 (32%)	10 (46%)
Normal, 5 (23%)	1 (20%)	3 (60%)	1 (20%)
Abnormal, 17 (77%)	4 (24%)	4 (24%)	9 (52%)
*BOLD right HC network*			
Total, 22 (100%)	5 (23%)	5 (23%)	12 (55%)
Normal, 4 (18%)	1 (25%)	2 (50%)	1 (25%)
Abnormal, 18 (82%)	4 (22%)	3 (17%)	11 (61%)

BOLD = blood-oxygen-level-dependent; DMN = default mode network; HC = hippocampal; MRI = magnetic resonance imaging; NBM = nucleus basalis of Meynert; rCBF = relative cerebral blood flow.

### 3.2. ROI group analysis

**Figure 2.**
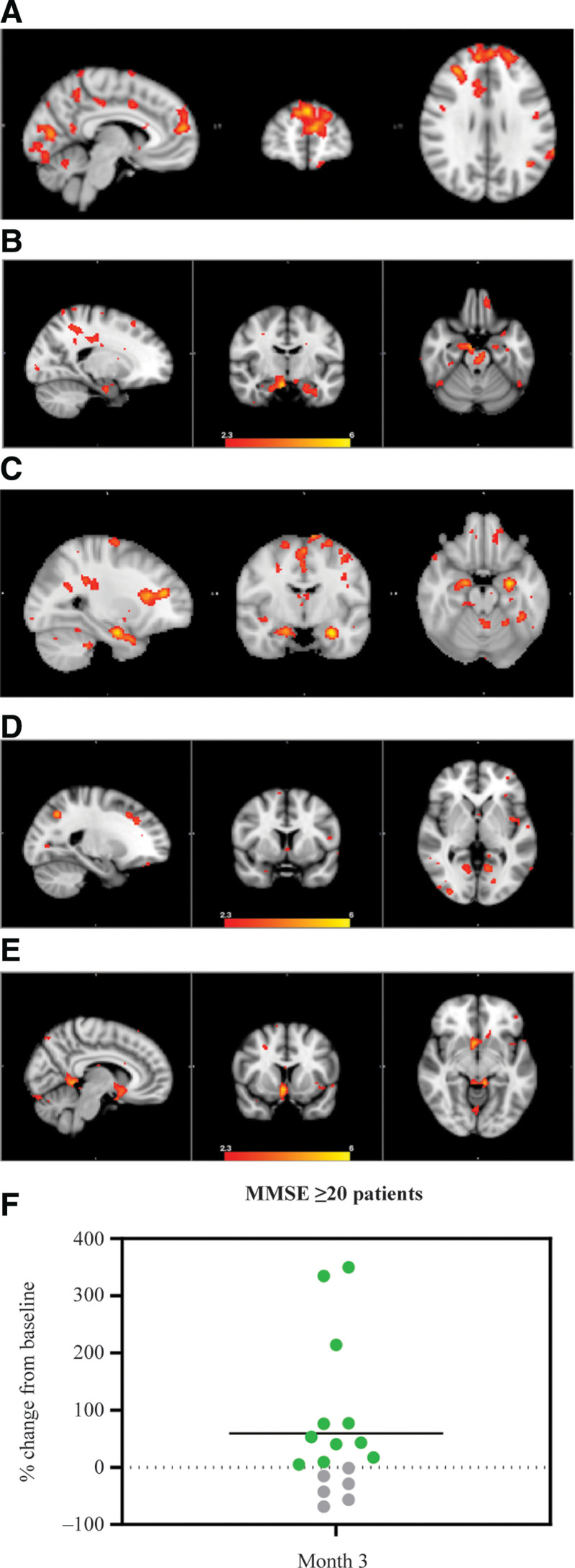
The neurophysiological health of patients before and after treatment with NE3107 was evaluated using advanced MRI of the brain. (A–E) Statistically significant regions of increased connectivity for (A) precuneus ROI (z-score > 3.1), (B) left HC ROI (z-score > 2.3), (C) right HC ROI (z-score > 2.3), (D) left NBM ROI (z-score > 2.3), and (E) right NBM ROI (z-score > 2.3); (F) Percent change from baseline in glutathione levels in the precuneus as measured by MRS (*P* = .069). HC = hippocampus; MMSE = Mini-Mental State Examination; MRI = magnetic resonance imaging; MRS = magnetic resonance spectroscopy; NBM = nucleus basalis of Meynert; ROI = region of interest.

With an ROI in the precuneus, there was a statistically significant (z-score > 2.3) increase in FC compared to other areas of the DMN, including the prefrontal cortex (Fig. 2A). With an ROI placed in either HC or NBM, there was a statistically significant increase in connectivity between the hippocampi and NBM, respectively (Fig. 2B–E).

### 3.3. Magnetic resonance spectroscopy

Brain glutathione levels in the precuneus were assessed using MRS. For patients with MCI or mild AD (MMSE ≥ 20), 11/17 (69%) improved, with a 59% increase in brain glutathione levels (Fig. 2F and Table 4).

**Table 4:** Summary of results from cognitive performance and biomarker assessments.

Endpoint	All patients					Patients with MMSE ≥ 20				
Cognitive assessment	Baseline, mean (95% CI)	Posttreatment, mean (95% CI)	Patients with improvement (%)	Mean change from baseline (95% CI)	*P* value*	Baseline, mean (95% CI)	Posttreatment, mean (95% CI)	Patients with improvement (%)	Mean change from baseline (95% CI)	*P* value*
ADAS-Cog11	15.28 (10.59, 19.96)	14.36 (8.74, 19.98)	13/23 (57)	–0.91 (–2.67, 0.84)	.29	10.50 (7.64, 13.36)	8.33 (5.51, 11.15)	13/18 (72)	–2.17 (–3.90,–0.43)	.017†
MMSE	24.00 (21.57, 26.43)	23.26 (20.39, 26.14)	8/23 (35)	–0.74 (–1.90, 0.42)	.20	26.44 (24.80, 28.09)	26.06 (24.12, 27.99)	8/18 (44)	–0.39 (–1.72, 0.95)	.55
MoCA	20.57 (17.59, 23.54)	20.52 (17.14, 23.91)	9/23 (39)	–0.04 (–0.96, 0.87)	.92	23.50 (21.44, 25.56)	24.06 (22.03, 26.08)	9/18 (50)	0.56 (–0.38, 1.49)	.23
QDRS	4.98 (3.74, 6.22)	4.44 (2.503, 6.37)	14/23 (61)	–0.54 (–1.77, 0.69)	.37	4.22 (3.16, 5.29)	2.67 (1.97, 3.36)	13/18 (72)	–1.56 (–2.46,–0.65)	.002†
CDR	0.61 (0.52, 0.70)	0.65 (0.41, 0.90)	4/23 (17)	0.04 (–0.14, 0.23)	.63	0.56 (0.48, 0.64)	0.44 (0.33, 0.56)	4/18 (22)	–0.11 (–0.22,–0.005)	.042†
ADCOMS	0.34 (0.22, 0.46)	0.35 (0.16, 0.53)	13/23 (57)	0.0049 (–0.079, 0.089)	.90	0.23 (0.16, 0.30)	0.17 (0.101, 0.21)	13/18 (72)	–0.07 (–0.12,–0.019)	.0094†

	All patients					Patients with MMSE ≥ 20				
Biomarker	Baseline, mean (95% CI)	Posttreatment, mean (95% CI)	Patients with improvement (%)	Mean change from baseline (95% CI)	*P* value*	Baseline, mean (95% CI)	Posttreatment, mean (95% CI)	Patients with improvement (%)	Mean change from baseline (95% CI)	*P* value*
Brain glutathione, mM	1.25 (0.92, 1.57)	1.35 (1.088, 1.62)	13/22 (59)	0.11 (–0.23, 0.45)	.54	1.12 (0.76, 1.48)	1.35 (1.051, 1.65)	12/18 (67)	0.23 (–0.15, 0.61)	.22
Plasma TNF-α, pg/mL	1.33 (0.73, 1.94)	0.93 (0.56, 1.304)	11/18 (61)	–0.45 (–1.22, 0.31)	.23	1.43 (0.67, 2.19)	0.91 (0.43, 1.402)	9/14 (64)	–0.56 (–1.55, 0.42)	.24
CSF P-tau:Aβ42 ratio	0.047 (0.0304, 0.063)	0.044 (0.028, 0.059)	10/16 (63)	–0.0033 (–0.008, 0.002)	.18	0.033 (0.019, 0.046)	0.0304 (0.019, 0.042)	7/11 (64)	–0.0024 (–0.005, 0.00)	.04†
CSF P-tau, pg/mL	31.87 (23.49, 40.25)	30.78 (22.72, 38.83)	13/21 (62)	–1.1 (–3.04, 0.85)	.26	26.46 (19.40, 33.52)	24.80 (18.49, 31.11)	10/16 (63)	–1.66 (–3.17,–0.14)	.033†
CSF Aβ42, pg/mL	842.3 (696.6, 987.9)	852.0 (709.2, 994.8)	11/18 (61)	9.72 (–60.72, 80.17)	.77	939.5 (770.6, 1108)	930.8 (750.8, 1111)	9/13 (69)	–8.69 (–80.95, 63.56)	.80

Aβ42 = amyloid beta peptide 42; ADAS-Cog11 = 11-item Alzheimer’s Disease Assessment Scale-Cognitive Subscale; ADCOMS = Alzheimer’s Disease Composite Score; CDR = Clinical Dementia Rating; CSF = cerebrospinal fluid; MMSE = Mini-Mental State Examination; MoCA = Montreal Cognitive Assessment; P-tau = phosphorylated tau protein; QDRS = Quick Dementia Rating Scale; TNF-α = tumor necrosis factor alpha.

* 1-way *t*-test.

† Statistical significance (*P* < .05).

### 3.4. Secondary outcomes: neuropsychological testing

**Figure 3.**
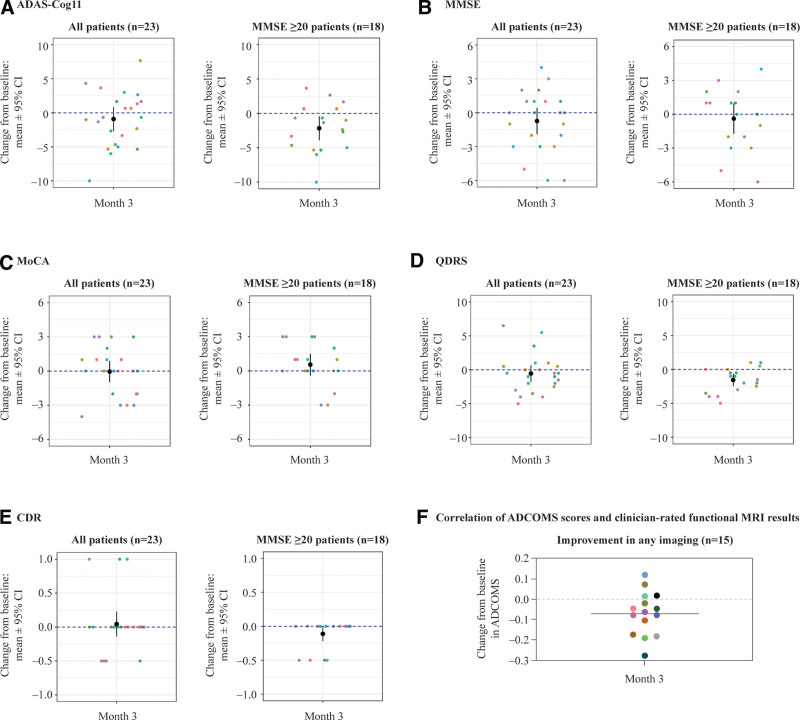
Secondary endpoints evaluated changes, after treatment with NE3107, in neuropsychological health using various cognitive assessments. (A–E) Change from baseline in (A) ADAS-Cog11 scores (all patients, *P* = .29; MMSE ≥ 20 patients, *P* = .017); (B) MMSE scores (all patients, *P* = .20; MMSE ≥ 20 patients, *P* = .55); (C) MoCA scores (all patients; *P* = .92; MMSE ≥ 20 patients, *P* = .23); (D) QDRS scores (all patients, *P* = .37; MMSE ≥ 20 patients, *P* = .002); (E) CDR scores (all patients, *P* = .63; MMSE ≥ 20 patients, *P* = .042). (F) Change from baseline in ADCOMS in patients with clinician-rated improvements in any fMRI analyses. ADAS-Cog11 = 11-item Alzheimer’s Disease Assessment Scale-Cognitive Subscale; ADCOMS = Alzheimer’s Disease Composite Score; CDR = Clinical Dementia Rating; fMRI = functional magnetic resonance imaging; MMSE = Mini-Mental State Examination; MoCA = Montreal Cognitive Assessment; QDRS = Quick Dementia Rating Scale.

The co-secondary endpoint of this study was to evaluate the effect of NE3107 treatment on neuropsychological health as assessed by cognitive performance testing administered at baseline and treatment completion using ADAS-Cog11, MMSE, MoCA, and ADCOMS. Fifty-seven percent (n = 13) of all 23 patients and 72% (n = 13) of 18 patients with MMSE ≥ 20 had an improved (lower) ADAS-Cog11 score at completion compared with baseline (Fig. 3A and Table 4). Thirty-five percent (n = 8) of all 23 patients and 44% (n = 8) of 18 patients with MMSE ≥ 20 had an improved (higher) MMSE score at completion compared with baseline (Fig. 3B and Table 4). Thirty-nine percent (n = 9) of all 23 patients and 50% (n = 9) of 18 patients with MMSE ≥ 20 had improved (higher) MoCA scores at completion compared with baseline (Fig. 3C and Table 4). Fifty-seven percent (n = 13) of all 23 patients and 72% (n = 13) of 18 patients with MMSE ≥ 20 had improved (lower) ADCOMS at completion compared with baseline (Table 4).

Neuropsychological effects of NE3107 were also measured by evaluating changes in the QDRS, as well as changes in QDRS-derived CDR levels. Sixty-one percent (n = 14) of all 23 patients and 72% (n = 13) of 18 patients with MMSE ≥ 20 had improved (lower) QDRS scores at treatment completion compared with baseline (Fig. 3D and Table 4). Additionally, 17% (n = 4) of all 23 patients and 22% (n = 4) of 18 patients with MMSE ≥ 20 had a reduction in CDR level at treatment completion compared with baseline, indicating improved cognitive function (Fig. 3E and Table 4).

Overall, NE3107 treatment was associated with statistically significant improvements in the clinician-reported (mean change: 1.7; *P* = .0038), patient-reported (mean change: 2.2; *P* < .001), and caretaker-reported (mean change: 1.07; *P* = .038) GRC (Table 5). Similarly, in patients with MMSE ≥ 20, NE3107 treatment was associated with statistically significant improvements in the clinician-reported (mean change: 2.67; *P* < .001), patient-reported (mean change: 2.08; *P* = .0012), and caretaker-reported (mean change: 1.69; *P* = .0011) GRC (Table 5).

**Table 5:** Summary of results from Global Rating of Change assessments.

GRC assessment	All patients (N = 23)		Patients with MMSE ≥ 20 (n = 18)	
	Mean (95% CI)	*P* value*	Mean (95% CI)	*P* value*
Clinician-reported	1.7 (0.61, 2.78)	.0038	2.67 (1.90, 3.43)	<.001
Patient-reported	2.2 (1.21, 3.18)	<.001	2.08 (0.95, 3.22)	.0012
Caretaker-reported	1.07 (0.067, 2.063)	.038	1.69 (0.78, 2.61)	.0011

CI = confidence interval; GRC = Global Rating of Change.

* 1-way *t*-test.

### 3.5. Correlation of neuroimaging and neuropsychological testing results

Improvements in ADCOMS were correlated with the clinician-rated imaging results for patients with MCI or mild AD (MMSE ≥ 20). For this group, an abnormal score on any of the functional imaging modalities (rCBF, DMN, HC networks, NBM) at baseline and improvement at follow-up was associated with improved ADCOMS (*P < *.05) (Fig. 3F).

### 3.6. Exploratory endpoints: biomarkers

The exploratory endpoints of this study investigated the anti-inflammatory and insulin-sensitizing effects of 20-mg oral NE3107 administered BID for 3 months; these effects were measured via serological biomarkers and cerebrospinal fluid (CSF) biomarkers.

### 3.7. Serological biomarker results

**Figure 4.**
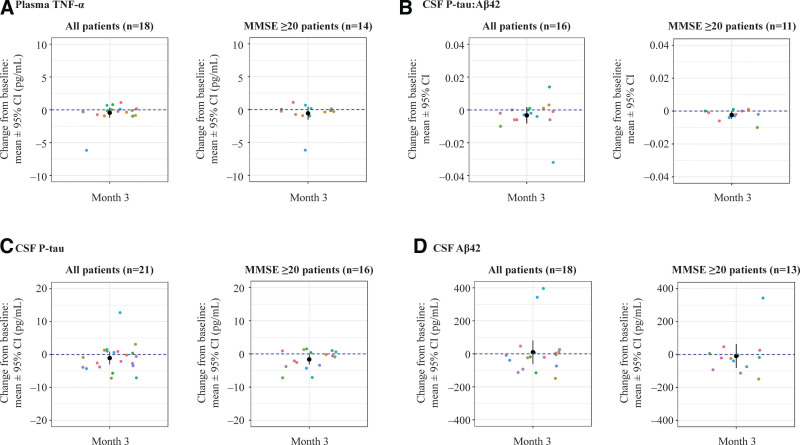
Exploratory endpoints assessed changes in biomarkers associated with neuroinflammation and AD in patients after treatment with NE3107. (A) Change from baseline in plasma TNF-α for all patients (*P* = .23) and patients with MMSE ≥ 20 (*P* = .24). (B–D) Change from baseline in CSF levels of B. P-tau:Aβ42 ratio (all patients, *P* = .18; MMSE ≥ 20 patients, *P* = .04); (C) P-tau levels (all patients, *P* = .26; MMSE ≥ 20 patients, *P* = .034); (D) Aβ42 levels (all patients, *P* = .77; MMSE ≥ 20 patients, *P* = .80). Aβ42 = amyloid beta peptide 42; CSF = cerebrospinal fluid; MMSE = Mini-Mental State Examination; P-tau = phosphorylated tau protein; TNF-α = tumor necrosis factor alpha.

Plasma TNF-α levels were analyzed at baseline and study completion in 18 out of 23 patients. Of these, 61% (n = 11) had reduced plasma TNF-α levels at study completion compared with baseline. Additionally, 64% (n = 9) of the 14 patients with MMSE ≥ 20 showed a reduction over the course of the 3-month treatment (Fig. 4A and Table 4). At baseline, only 4% (n = 1) of the study patients were diabetic, and hence any apparent effects of NE3107 on glucose metabolism were not ascertained in this study.

### 3.8. CSF biomarker results

Patients with MCI and AD have been shown to have lower CSF Aβ42 levels and increased CSF P-tau levels compared to cognitively unimpaired individuals.^[27–30]^ In this study, 63% (n = 10) of 16 total patients and 64% (n = 7) of 11 patients with MMSE ≥ 20 had a lower P-tau:Aβ42 ratio at study completion compared with baseline (Fig. 4B and Table 4). CSF P-tau levels were reduced in 62% (n = 13) of 21 patients and 63% (n = 10) of 16 patients with MMSE ≥ 20, compared with baseline (Fig. 4C and Table 4). CSF Aβ42 levels decreased in 61% (n = 11) of 18 total patients and in 69% (n = 9) of 13 patients with MMSE ≥ 20, compared with baseline (Fig. 4D and Table 4).

### 3.9. Multimodal correlation analyses

#### 3.9.1. Correlations between changes in cognitive performance and biomarkers

Since AD is thought to be a multifactorial disease,^[31]^ we sought to demonstrate correlations among improvements in cognitive performance and various biomarkers, as well as between different cognitive performance or biomarker assessments.

For the total patient population, we observed statistically significant correlations between posttreatment improvements in QDRS scores and ADCOMS (*r* = 0.91; *P < *.001); ADAS-Cog11 scores and brain glutathione levels (*r* = −0.45; *P = *.034); ADCOMS and Aβ42 levels (*r* = 0.53; *P = *.025); ADCOMS and P-tau levels (*r* = 0.49; *P = *.024); Aβ42 and P-tau levels (*r* = 0.72; *P = *.0016); Aβ42 levels and the P-tau:Aβ42 ratio (*r* = −0.71; *P = *.0021); and P-tau levels and the P-tau:Aβ42 ratio (*r* = −0.59; *P* = .016) (Table S1, Supplemental Digital Content, http://links.lww.com/MD/N249). We also observed statistically non-significant correlations between posttreatment improvements from baseline in ADAS-Cog11 scores and TNF-α levels (*r* = 0.46; *P = *.054) and TNF-α and brain glutathione levels (*r* = −0.44; *P* = .076).

For patients with MCI to mild dementia (MMSE ≥ 20; n = 18), we found statistically significant correlations between posttreatment improvements in ADAS-Cog11 scores and TNF-α levels (*r* = 0.59; *P* = .026); QDRS scores and ADCOMS (*r* = 0.76; *P* < .001); and P-tau levels and the P-tau:Aβ42 ratio (*r* = 0.74; *P = *.0086) (Table S1, Supplemental Digital Content, http://links.lww.com/MD/N249). We also observed statistically non-significant correlations between posttreatment improvements from baseline in ADCOMS and brain glutathione levels (*r* = 0.45; *P = *.059); TNF-α and brain glutathione levels (*r* = −0.53; *P = *.05); and Aβ42 and brain glutathione levels (*r* = 0.50; *P = *.084).

## 4. Discussion

Old age is the greatest risk factor for AD, and expanding geriatric populations are likely to exacerbate the already substantial healthcare costs associated with AD. Hence, there is an urgent medical need for drugs that can alter the progression of AD.^[32,33]^ In the present phase 2, open-label, exploratory study, we evaluated the potential scope of treatment-related improvements in patients with mild-to-moderate dementia using neuroimaging endpoints (neurophysiology), cognitive performance tests (neuropsychology), and biomarkers of AD pathology, inflammation, and oxidative stress following treatment with the anti-inflammatory and insulin-sensitizing agent, NE3107. Overall, treatment with 20 mg oral NE3107 BID for 3 months appeared to be associated with improvements in neurophysiology, oxidative stress, cognition, and biomarkers related to neuroinflammation and AD. Importantly, we demonstrated statistically significant correlations among improvements in neuroimaging endpoints, cognitive performance, and AD-related biomarkers (Table S1, Supplemental Digital Content, http://links.lww.com/MD/N249).

NE3107 was originally found while screening for synthetic analogs of a naturally occurring dehydroepiandrosterone derivative with potent anti-inflammatory properties.^[34,35]^ NE3107 is thought to bind to ERK and prevent its activation within specific signaling complexes associated only with pathological inflammation, without perturbing ERK’s homeostatic activity, which is mediated by the Ras-Raf-MEK-ERK pathway. NE3107 inhibits downstream signal transduction events involving NF-κB that typically lead to pro-inflammatory responses, apoptosis, and insulin resistance.^[36,37]^ In murine models of arthritis, NE3107 was associated with anti-inflammatory effects, such as reduced production of several key inflammatory mediators (e.g., TNF-α and IL-6), without being immune suppressive.^[34,38]^ TNF-α is thought to play a central role in neuroinflammation and AD pathogenesis, and suppressing this pro-inflammatory mediator may improve cognition and slow AD-associated cognitive decline, as demonstrated by improvements in brain pathology and cognitive function in rodent AD models after treatment with anti-TNF-α therapies. Moreover, patients with rheumatoid arthritis or psoriasis who were taking anti-TNF therapies showed a decreased risk of developing AD.^[14,32,39]^ Indeed, neuroinflammation is believed to be a founding event in AD, perhaps occuring even prior to Aβ plaque deposition.^[40]^ Consistent with these findings, we showed that NE3107 treatment was associated with marked, albeit statistically non-significant, reductions in plasma TNF-α levels from baseline. Statistically significant correlations among improvements in neuroimaging endpoints, cognitive performance, and AD-related biomarkers support this hypothesis and should be further studied (Table S1, Supplemental Digital Content, http://links.lww.com/MD/N249).

Oxidative stress is thought to play an important role in the development of MCI and AD, and reduced CNS glutathione, an antioxidant buffer in the brain, has been implicated in AD-related neurodegeneration.^[8]^ Additionally, reduced brain glutathione levels were significantly correlated with diminished cognitive function, as measured by MMSE and CDR, in patients with MCI and AD.^[41]^ Inflammation and oxidative stress are mutually exacerbating; specifically, TNF-α, via the activation of the MAPK and NF-κB pathway, can increase reactive oxygen species levels and reduce glutathione levels.^[6,7]^ In turn, depletion of intracellular glutathione can induce a neuroinflammatory response involving NF-κB and ERK in microglia and astrocytes, leading to the release of pro-inflammatory cytokines, such as TNF-α and IL-6.^[42]^ Consistent with the anti-inflammatory effects of NE3107, we demonstrated an increase in brain glutathione levels in patients with MCI or dementia after 3 months of NE3107 treatment. Moreover, we found statistically significant correlations between increases in brain glutathione and reductions in plasma TNF-α (indicating decreased inflammation; *r* = −0.53; *P = *.05) in patients with MCI or mild dementia (MMSE ≥ 20) and reductions in ADAS-Cog11 scores (indicating improved cognition; *r* = −0.45; *P = *.034) in all 23 patients.

P-tau and Aβ42 plaques are considered to be pathological hallmarks of AD and are pro-inflammatory, forming feedback loops with neuroinflammation in AD pathogenesis.^[4,5,43]^ Moreover, they can be detected several years prior to the onset of clinical symptoms of AD,^[40,44]^ and the CSF ratio of P-tau:Aβ42 (elevated levels of P-tau and low levels of Aβ42) was shown to be a preclinical and predictive biomarker of cognitive impairment and dementia.^[45]^ In line with the anti-TNF-α effect of NE3107, we saw statistically significant reductions in CSF P-tau levels and the P-tau:Aβ42 ratio in patients with baseline MMSE ≥ 20. Additionally, we noted a statistically significant correlation (*r* = 0.49; *P = *.024) between changes from baseline in the CSF P-tau level and ADCOMS.

The predefined hypotheses of this study were based on the previously demonstrated anti-inflammatory and insulin-sensitizing properties of NE3107. Accordingly, the primary objective of this study was to demonstrate improvements in the neurophysiology (using neuroimaging) of patients after treatment with NE3107. After 3 months of treatment, NE3107 was descriptively associated with clinician-rated, statistically significant improvements from baseline in rCBF within the temporo-parieto-occipital lobe (measured using ASL) and FC (measured using BOLD imaging) within the DMN, as well as the NBM and HC networks. Present in the parietal lobe, the precuneus has integral functions within the DMN and is important for episodic and autobiographical memory. Consistent with its importance, the precuneus is thought to consume more glucose than any other region of the human brain, and compromised CBF activity within this region may be associated with AD progression.^[39]^ ASL-MRI-derived changes in perfusion strongly correlate with changes in glucose metabolism measured using fluorodeoxyglucose positron emission tomography. Thus, improvements in perfusion (rCBF) are expected to correspond to improvements in brain glucose and energy utilization.^[46–48]^

Our study had several limitations. First, our sample size, while appropriate for an exploratory study, was relatively small, and the duration of active treatment was short. Despite the short duration, several of the observed effects associated with NE3107 for the primary and secondary endpoints were in the direction of therapeutic benefit. Second, there was a lack of peripheral glycemic dysregulation in the study patients—only 4% (n = 1) of the study patients were diabetic—precluding any apparent effects of NE3107 on glucose metabolism in this study. Additionally, our study did not employ any surrogate endpoints to measure changes in insulin signaling in the CNS of the study patients. Fourth, the open-label study design limited our ability to isolate potential placebo effects. However, this study served as an exploratory precursor to inform the design of subsequent larger, placebo-controlled confirmatory trials. Fifth, given the exploratory nature of this study, we utilized multiple comparisons to gain an understanding of the scope of treatment effects associated with NE3107, which may have affected the accuracy of the conclusions. Lastly, we acknowledge a lack of ethnic and racial diversity in our study patients. Future studies with NE3107 will aim to include racially and ethnically diverse groups of patients.

An important finding of this study was that patients with MCI or mild dementia (MMSE ≥ 20 at baseline) showed greater improvements in several endpoints (Figs. 3 and 4 and Table 4), suggesting that the potential extent of the therapeutic effects of NE3107 may be more pronounced in patients who are in the prodromal or early stages of dementia. Compelling evidence suggests that AD progression may follow a more intricate, rather than unidirectional, path, its pathophysiology comprising several aberrant positive feedback loops that may form several years prior to disease manifestation.^[40,44,49]^ Therefore, early intervention with an immunomodulatory, and possibly, multipotent, therapeutic such as NE3107 may be a valuable line of defense and could alter the clinical course of AD.^[49,50]^ Subsequent longer-term, placebo-controlled studies are required to validate the potential of NE3107 in patients in the early stages of dementia.

## Acknowledgments

Medical writing, editing, and publication assistance were provided by *P*-value communications.

## Author contributions

**Conceptualization:** Kennedy Mahdavi, Clarence Ahlem, Christopher Reading, Joseph Palumbo, Bijan Pourat, Taylor Kuhn, Sheldon Jordan.

**Data curation:** Jonathan Haroon, Kaya Jordan, Kennedy Mahdavi, Elisabeth Rindner, Sergio Becerra, Jean Rama Surya, Margaret Zielinski.

**Formal analysis:** Dayan Goodenowe, Kaitlyn Hofmeister, Jeffrey Zhang, Taylor Kuhn.

**Funding acquisition:** Victoria Venkatraman.

**Investigation:** Jonathan Haroon, Kaya Jordan, Kennedy Mahdavi, Elisabeth Rindner, Sergio Becerra, Jean Rama Surya, Margaret Zielinski.

**Methodology:** Clarence Ahlem, Christopher Reading, Joseph Palumbo, Sheldon Jordan.

**Project administration:** Jonathan Haroon, Kennedy Mahdavi, Margaret Zielinski.

**Resources:** Dayan Goodenowe, Kaitlyn Hofmeister, Jeffrey Zhang, Joseph Palumbo, Sheldon Jordan.

**Software:** Sergio Becerra.

**Supervision:** Joseph Palumbo, Taylor Kuhn, Sheldon Jordan.

**Validation:** Clarence Ahlem, Christopher Reading, Joseph Palumbo.

**Visualization:** Jonathan Haroon, Kaya Jordan, Kennedy Mahdavi, Elisabeth Rindner, Sergio Becerra, Jean Rama Surya, Margaret Zielinski, Victoria Venkatraman, Clarence Ahlem, Christopher Reading, Joseph Palumbo, Sheldon Jordan.

**Writing – original draft:** Jonathan Haroon, Kaya Jordan, Kennedy Mahdavi, Elisabeth Rindner, Sergio Becerra, Jean Rama Surya, Margaret Zielinski, Victoria Venkatraman, Clarence Ahlem, Christopher Reading, Joseph Palumbo, Sheldon Jordan.

**Writing – review & editing:** Jonathan Haroon, Kaya Jordan, Kennedy Mahdavi, Elisabeth Rindner, Sergio Becerra, Jean Rama Surya, Margaret Zielinski, Victoria Venkatraman, Clarence Ahlem, Christopher Reading, Joseph Palumbo, Sheldon Jordan.

## Supplementary Material

**Figure:**
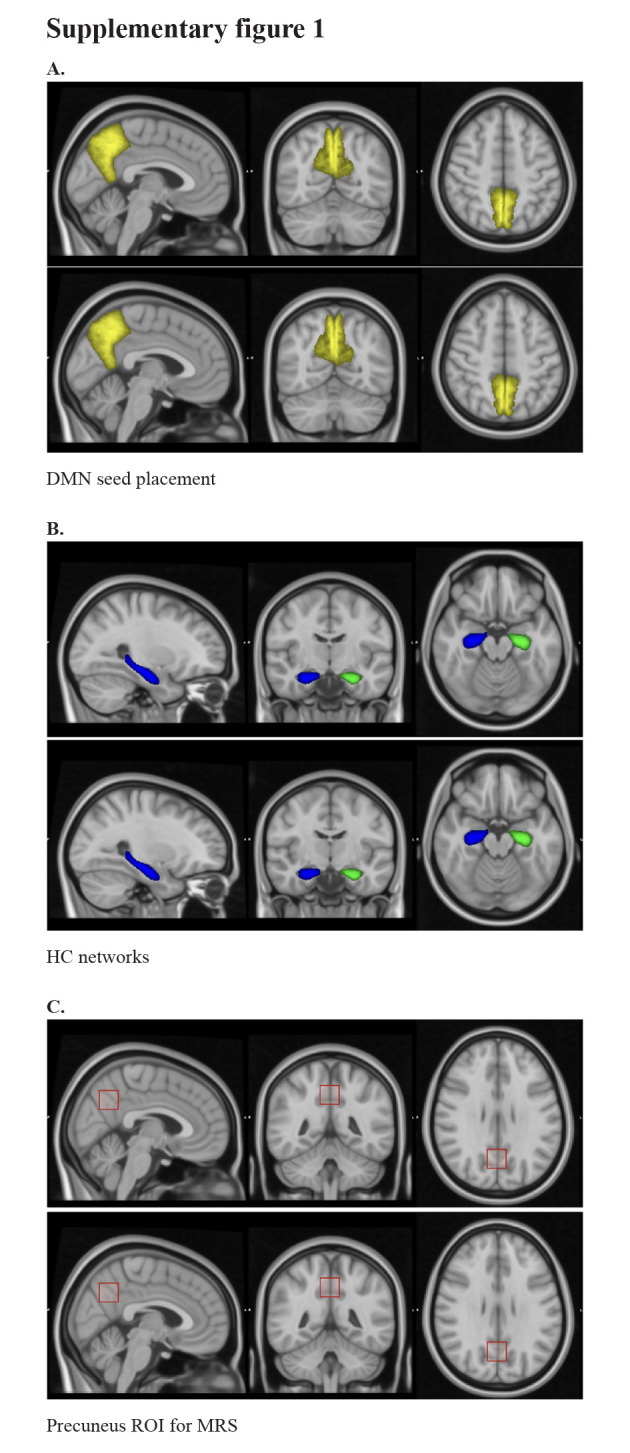


## References

[1] Kinney JW Bemiller S Murtishaw AS Leisgang AM Salazar AM Lamb BT. Inflammation as a central mechanism in Alzheimer’s disease. Alzheimers Dement (NY). 2018;4:575–90. 10.1016/j.trci.2018.06.014 PMC6214864 30406177

[2] Rorbach-Dolata A Piwowar A. Neurometabolic evidence supporting the hypothesis of increased incidence of type 3 diabetes mellitus in the 21st century. Biomed Res Int. 2019;2019:1435276. 31428627 10.1155/2019/1435276 PMC6679855

[3] Jung YJ Tweedie D Scerba MT Greig NH. Neuroinflammation as a factor of neurodegenerative disease: thalidomide analogs as treatments. Front Cell Dev Biol. 2019;7:313. 31867326 10.3389/fcell.2019.00313 PMC6904283

[4] Wei Z Koya J Reznik SE. Insulin resistance exacerbates Alzheimer disease via multiple mechanisms. Front Neurosci. 2021;15:687157. 34349617 10.3389/fnins.2021.687157 PMC8326507

[5] Reading CL Ahlem CN Murphy MF. NM101 Phase III study of NE3107 in Alzheimer’s disease: rationale, design and therapeutic modulation of neuroinflammation and insulin resistance. Neurodegener Dis Manag. 2021;11:289–98. 34251287 10.2217/nmt-2021-0022

[6] Kim JJ Lee SB Park JK Yoo YD. TNF-alpha-induced ROS production triggering apoptosis is directly linked to Romo1 and Bcl-X(L). Cell Death Differ. 2010;17:1420–34. 20203691 10.1038/cdd.2010.19

[7] Wu L Pan Y. Reactive oxygen species mediate TNF-alpha-induced inflammatory response in bone marrow mesenchymal cells. Iran J Basic Med Sci. 2019;22:1296–301. 32128094 10.22038/ijbms.2019.37893.9006 PMC7038432

[8] Chen JJ Thiyagarajah M Song J. Altered central and blood glutathione in Alzheimer’s disease and mild cognitive impairment: a meta-analysis. Alzheimers Res Ther. 2022;14:23. 35123548 10.1186/s13195-022-00961-5 PMC8818133

[9] Draznin B. Molecular mechanisms of insulin resistance: serine phosphorylation of insulin receptor substrate-1 and increased expression of p85alpha: the two sides of a coin. Diabetes. 2006;55:2392–7. 16873706 10.2337/db06-0391

[10] Zhang Q Guo S Zhang X. Amyloid beta oligomer-induced ERK1/2-dependent serine 636/639 phosphorylation of insulin receptor substrate-1 impairs insulin signaling and glycogen storage in human astrocytes. Gene. 2015;561:76–81. 25667991 10.1016/j.gene.2015.02.011

[11] Berlanga-Acosta J Guillen-Nieto G Rodriguez-Rodriguez N. Insulin resistance at the crossroad of Alzheimer disease pathology: a review. Front Endocrinol (Lausanne). 2020;11:560375. 33224105 10.3389/fendo.2020.560375 PMC7674493

[12] Holscher C. Brain insulin resistance: role in neurodegenerative disease and potential for targeting. Expert Opin Investig Drugs. 2020;29:333–48. 10.1080/13543784.2020.1738383 32175781

[13] Chow HM Shi M Cheng A. Age-related hyperinsulinemia leads to insulin resistance in neurons and cell-cycle-induced senescence. Nat Neurosci. 2019;22:1806–19. 31636448 10.1038/s41593-019-0505-1

[14] Zhou M Xu R Kaelber DC Gurney ME. Tumor Necrosis Factor (TNF) blocking agents are associated with lower risk for Alzheimer’s disease in patients with rheumatoid arthritis and psoriasis. PLoS One. 2020;15:e0229819. 32203525 10.1371/journal.pone.0229819 PMC7089534

[15] Wang T Villegas S Huang Y. Amelioration of glucose intolerance by the synthetic androstene HE3286: link to inflammatory pathways. J Pharmacol Exp Ther. 2010;333:70–80. 20068030 10.1124/jpet.109.161182

[16] Lu M Patsouris D Li P. A new antidiabetic compound attenuates inflammation and insulin resistance in Zucker diabetic fatty rats. Am J Physiol Endocrinol Metab. 2010;298:E1036–1048. 20159859 10.1152/ajpendo.00668.2009 PMC2867370

[17] Reading CL Flores-Riveros J Stickney DR Frincke JM. An anti-inflammatory sterol decreases obesity-related inflammation-induced insulin resistance and metabolic dysregulation. Mediators Inflamm. 2013;2013:814989. 23431246 10.1155/2013/814989 PMC3572652

[18] Reading CL Stickney DR Flores-Riveros J. A synthetic anti-inflammatory sterol improves insulin sensitivity in insulin-resistant obese impaired glucose tolerance subjects. Obesity (Silver Spring). 2013;21:E343–349. 23670958 10.1002/oby.20207

[19] Kuhn T Becerra S Duncan J. Translating state-of-the-art brain magnetic resonance imaging (MRI) techniques into clinical practice: multimodal MRI differentiates dementia subtypes in a traditional clinical setting. Quant Imaging Med Surg. 2021;11:4056–73. 34476189 10.21037/qims-20-1355 PMC8339641

[20] Galvin JE. The Quick Dementia Rating System (Qdrs): a rapid dementia staging tool. Alzheimers Dement (Amst). 2015;1:249–59. 26140284 10.1016/j.dadm.2015.03.003 PMC4484882

[21] Schrag A Schott JM; Alzheimer's Disease Neuroimaging Initiative. What is the clinically relevant change on the ADAS-Cog? J Neurol Neurosurg Psychiatry. 2012;83:171–3. 22019547 10.1136/jnnp-2011-300881

[22] Shigemori K Ohgi S Okuyama E Shimura T Schneider E. The factorial structure of the Mini-Mental State Examination (MMSE) in Japanese dementia patients. BMC Geriatr. 2010;10:36. 20534132 10.1186/1471-2318-10-36 PMC2903593

[23] Breton A Casey D Arnaoutoglou NA. Cognitive tests for the detection of mild cognitive impairment (MCI), the prodromal stage of dementia: meta-analysis of diagnostic accuracy studies. Int J Geriatr Psychiatry. 2019;34:233–42. 30370616 10.1002/gps.5016

[24] Nasreddine ZS Phillips NA Bedirian V. The montreal cognitive assessment, MoCA: a brief screening tool for mild cognitive impairment. J Am Geriatr Soc. 2005;53:695–9. 15817019 10.1111/j.1532-5415.2005.53221.x

[25] Wang J Logovinsky V Hendrix SB. ADCOMS: a composite clinical outcome for prodromal Alzheimer’s disease trials. J Neurol Neurosurg Psychiatry. 2016;87:993–9. 27010616 10.1136/jnnp-2015-312383 PMC5013117

[26] Kamper SJ Maher CG Mackay G. Global rating of change scales: a review of strengths and weaknesses and considerations for design. J Man Manip Ther. 2009;17:163–70. 20046623 10.1179/jmt.2009.17.3.163 PMC2762832

[27] Ibach B Binder H Dragon M. Cerebrospinal fluid tau and beta-amyloid in Alzheimer patients, disease controls and an age-matched random sample. Neurobiol Aging. 2006;27:1202–11. 16085339 10.1016/j.neurobiolaging.2005.06.005

[28] Sunderland T Linker G Mirza N. Decreased beta-amyloid1-42 and increased tau levels in cerebrospinal fluid of patients with Alzheimer disease. JAMA. 2003;289:2094–103. 12709467 10.1001/jama.289.16.2094

[29] Hansson O Zetterberg H Buchhave P Londos E Blennow K Minthon L. Association between CSF biomarkers and incipient Alzheimer’s disease in patients with mild cognitive impairment: a follow-up study. Lancet Neurol. 2006;5:228–34. 16488378 10.1016/S1474-4422(06)70355-6

[30] Mattsson N Zetterberg H Hansson O. CSF biomarkers and incipient Alzheimer disease in patients with mild cognitive impairment. JAMA. 2009;302:385–93. 19622817 10.1001/jama.2009.1064

[31] Iqbal K Grundke-Iqbal I. Alzheimer’s disease, a multifactorial disorder seeking multitherapies. Alzheimers Dement. 2010;6:420–4. 20813343 10.1016/j.jalz.2010.04.006 PMC2946155

[32] Torres-Acosta N O’Keefe JH O’Keefe EL Isaacson R Small G. Therapeutic potential of TNF-alpha inhibition for Alzheimer’s disease prevention. J Alzheimers Dis. 2020;78:619–26. 33016914 10.3233/JAD-200711 PMC7739965

[33] Graff-Radford J Kantarci K. Magnetic resonance spectroscopy in Alzheimer’s disease. Neuropsychiatr Dis Treat. 2013;9:687–96. 23696705 10.2147/NDT.S35440 PMC3658533

[34] Auci D Kaler L Subramanian S. A new orally bioavailable synthetic androstene inhibits collagen-induced arthritis in the mouse: androstene hormones as regulators of regulatory T cells. Ann N Y Acad Sci. 2007;1110:630–40. 17911478 10.1196/annals.1423.066

[35] Ahlem C Auci D Mangano K. HE3286: a novel synthetic steroid as an oral treatment for autoimmune disease. Ann N Y Acad Sci. 2009;1173:781–90. 19758229 10.1111/j.1749-6632.2009.04798.x

[36] Reading CL Frincke JM White SK. Molecular targets for 17alpha-ethynyl-5-androstene-3beta,7beta,17beta-triol, an anti-inflammatory agent derived from the human metabolome. PLoS One. 2012;7:e32147. 22384159 10.1371/journal.pone.0032147 PMC3286445

[37] Manzoor Z Koh Y-S. Mitogen-activated protein kinases in inflammation. J Bacteriol Virol. 2012;42:189.

[38] Auci DL Mangano K Destiche D. Oral treatment with HE3286 ameliorates disease in rodent models of rheumatoid arthritis. Int J Mol Med. 2010;25:625–33. 20198312 10.3892/ijmm_00000385

[39] Kucikova L Goerdten J Dounavi ME. Resting-state brain connectivity in healthy young and middle-aged adults at risk of progressive Alzheimer’s disease. Neurosci Biobehav Rev. 2021;129:142–53. 34310975 10.1016/j.neubiorev.2021.07.024

[40] Rodriguez-Vieitez E Saint-Aubert L Carter SF. Diverging longitudinal changes in astrocytosis and amyloid PET in autosomal dominant Alzheimer’s disease. Brain. 2016;139(Pt 3):922–36. 26813969 10.1093/brain/awv404 PMC4766380

[41] Mandal PK Saharan S Tripathi M Murari G. Brain glutathione levels—a novel biomarker for mild cognitive impairment and Alzheimer’s disease. Biol Psychiatry. 2015;78:702–10. 26003861 10.1016/j.biopsych.2015.04.005

[42] Lee M Cho T Jantaratnotai N Wang YT McGeer E McGeer PL. Depletion of GSH in glial cells induces neurotoxicity: relevance to aging and degenerative neurological diseases. FASEB J. 2010;24:2533–45. 20228251 10.1096/fj.09-149997

[43] Li X Li TQ Andreasen N Wiberg MK Westman E Wahlund LO. Ratio of Abeta42/P-tau181p in CSF is associated with aberrant default mode network in AD. Sci Rep. 2013;3:1339. 23439248 10.1038/srep01339 PMC3581831

[44] Arastoo M Lofthouse R Penny LK. Current progress and future directions for tau-based fluid biomarker diagnostics in Alzheimer’s disease. Int J Mol Sci . 2020;21:8673. 33212983 10.3390/ijms21228673 PMC7698492

[45] Fagan AM Roe CM Xiong C Mintun MA Morris JC Holtzman DM. Cerebrospinal fluid tau/beta-amyloid(42) ratio as a prediction of cognitive decline in nondemented older adults. Arch Neurol. 2007;64:343–9. 17210801 10.1001/archneur.64.3.noc60123

[46] Chen Y Wolk DA Reddin JS. Voxel-level comparison of arterial spin-labeled perfusion MRI and FDG-PET in Alzheimer disease. Neurology. 2011;77:1977–85. 22094481 10.1212/WNL.0b013e31823a0ef7 PMC3235355

[47] Wolk DA Detre JA. Arterial spin labeling MRI: an emerging biomarker for Alzheimer’s disease and other neurodegenerative conditions. Curr Opin Neurol. 2012;25:421–8. 22610458 10.1097/WCO.0b013e328354ff0a PMC3642866

[48] Verfaillie SC Adriaanse SM Binnewijzend MA. Cerebral perfusion and glucose metabolism in Alzheimer’s disease and frontotemporal dementia: two sides of the same coin? Eur Radiol. 2015;25:3050–9. 25899416 10.1007/s00330-015-3696-1 PMC4562004

[49] Doig AJ. Positive feedback loops in Alzheimer’s disease: the Alzheimer’s feedback hypothesis. J Alzheimers Dis. 2018;66:25–36. 30282364 10.3233/JAD-180583 PMC6484277

[50] Zilka N Kazmerova Z Jadhav S. Who fans the flames of Alzheimer’s disease brains? Misfolded tau on the crossroad of neurodegenerative and inflammatory pathways. J Neuroinflammation. 2012;9:47. 22397366 10.1186/1742-2094-9-47 PMC3334709

