# Supplementary material for: A phase 2, open-label study of anti-inflammatory NE3107 in patients with dementias
Source: Medicine (Baltimore). 2024 Jul 26;103(30):e39027. doi: 10.1097/MD.0000000000039027 (PMC11272329; doi:10.1097/MD.0000000000039027)
Supplement: Supplementary file 1 [file medi-103-e39027-s002.docx]

**Supplementary table 1. Multi-modal correlation analyses**

|  | **All patients**  **(N = 23)** | **Patients with MMSE ≥20**  **(n = 18)** |  |
| --- | --- | --- | --- |
| ***ADAS-Cog11 scores*** | | | |
| Plasma TNF-α | r = 0.46 (*P* = .054) | r = 0.59 (*P* = .026) |  |
| Brain glutathione | r = −0.45 (*P* = .034) | r = −0.32 (*P* = .20) |  |
| ***Total QDRS score*** | | | |
| ADCOMS | r = 0.91 (*P* < .001) | r = 0.76 (*P* < .001) |  |
| ***ADCOMS*** | | | |
| CSF Aβ42 | r = 0.53 (*P* = .025) | r = 0.46 (*P* = .11) |  |
| CSF P-tau | r = 0.49 (*P* = .024) | r = 0.37 (*P* = .16) |  |
| Brain glutathione | r = 0.0081 (*P* = .72) | r = 0.45 (*P* = .059) |  |
| ***CSF Aβ42*** | | |  |
| CSF P-tau | r = 0.72 (*P* = .0016) | r = 0.34 (*P* = .30) | |
| CSF Aβ42:P-tau ratio | r = −0.71 (*P* = .0021) | r = −0.20 (*P* = .55) | |
| Brain glutathione | r = 0.42 (*P* = .097) | r = 0.50 (*P* = .084) | |
| ***CSF P-tau*** | | | |
| CSF Aβ42:P-tau ratio | r = −0.59 (*P* = .016) | r = 0.74 (*P* = .0086) | |
| ***Plasma TNF-α*** | | | |
| Brain glutathione | r = −0.44 (*P* = .076) | r = −0.53 (*P* =.05) | |

r = correlation coefficient.
